# In vivo assessment of Lewy body and beta-amyloid copathologies in idiopathic normal pressure hydrocephalus: prevalence and associations with clinical features and surgery outcome

**DOI:** 10.1186/s12987-022-00368-2

**Published:** 2022-09-07

**Authors:** Giulia Giannini, Simone Baiardi, Sofia Dellavalle, Corrado Zenesini, Sabina Cevoli, Nils Danner, Henna-Kaisa Jyrkkänen, Marcello Rossi, Barbara Polischi, Corinne Quadalti, Camilla Stefanini, Pietro Cortelli, David Milletti, Sanna-Kaisa Herukka, Giorgio Palandri, Ville Leinonen, Piero Parchi

**Affiliations:** 1 IRCCS Istituto delle Scienze Neurologiche di Bologna, Bellaria Hospital, Via Altura 1/8, 40139 Bologna, Italy; 2 Department of Biomedical and Neuromotor Sciences (DIBINEM), University of Bologna, Bologna, Italy; 3 Department of Experimental, Diagnostic and Specialty Medicine (DIMES), University of Bologna, Bologna, Italy; 4 Department of Neurosurgery, Kuopio University Hospital, 70029 Kuopio, Finland; 5 Neurosurgery, Institute of Clinical Medicine, University of Eastern Finland, 70210 Kuopio, Finland; 6 Department of Neurology, Kuopio University Hospital, 70029 Kuopio, Finland; 7 Neurology, Institute of Clinical Medicine, University of Eastern Finland, 70210 Kuopio, Finland

**Keywords:** Idiopathic normal pressure hydrocephalus, Biomarkers, Cerebrospinal fluid, Surgery outcome, Movement disorders, RT-QuIC, Real-time quaking-induced conversion assay, α-synuclein, Lewy body

## Abstract

**Background:**

Idiopathic normal pressure hydrocephalus (iNPH) is a clinico-radiological syndrome of elderly individuals likely sustained by different neurodegenerative changes as copathologies. Since iNPH is a potentially reversible condition, assessing neurodegenerative pathologies in vitam through CSF biomarkers and their influence on clinical features and surgical outcome represents crucial steps.

**Methods:**

We measured α-synuclein seeding activity related to Lewy body (LB) pathology by the real-time quaking-induced conversion assay (RT-QuIC) and Alzheimer disease core biomarkers (proteins total-tau, phospho-tau, and amyloid-beta) by immunoassays in the cerebrospinal fluid (CSF) of 293 iNPH patients from two independent cohorts. To compare the prevalence of LB copathology between iNPH participants and a control group representative of the general population, we searched for α-synuclein seeding activity in 89 age-matched individuals who died of Creutzfeldt-Jakob disease (CJD). Finally, in one of the iNPH cohorts, we also measured the CSF levels of neurofilament light chain protein (NfL) and evaluated the association between all CSF biomarkers, baseline clinical features, and surgery outcome at 6 months.

**Results:**

Sixty (20.5%) iNPH patients showed α-synuclein seeding activity with no significant difference between cohorts. In contrast, the prevalence observed in CJD was only 6.7% (p = 0.002). Overall, 24.0% of iNPH participants showed an amyloid-positive (A+) status, indicating a brain co-pathology related to Aβ deposition. At baseline, in the Italian cohort, α-synuclein RT-QuIC positivity was associated with higher scores on axial and upper limb rigidity (p = 0.003 and p = 0.011, respectively) and lower MMSEc scores (p = 0.003). A+ patients showed lower scores on the MMSEc (p = 0.037) than A- patients. Higher NfL levels were also associated with lower scores on the MMSEc (rho = -0.213; p = 0.021). There were no significant associations between CSF biomarkers and surgical outcome at 6 months (i.e. responders defined by decrease of 1 point on the mRankin scale)**.**

**Conclusions:**

Prevalent LB- and AD-related neurodegenerative pathologies affect a significant proportion of iNPH patients and contribute to cognitive decline (both) and motor impairment (only LB pathology) but do not significantly influence the surgical outcome at 6 months. Their effect on the clinical benefit after surgery over a more extended period remains to be determined.

**Supplementary Information:**

The online version contains supplementary material available at 10.1186/s12987-022-00368-2.

## Background

Idiopathic normal pressure hydrocephalus (iNPH) is a potentially reversible clinical entity characterized by enlarged ventricles disproportionate to the degree of cortical atrophy on neuroimaging in patients without a clear etiology. Patients with iNPH suffer from a classical clinical triad of gait disturbance, urinary incontinence, and cognitive impairment, although some manifest only one or two of these symptoms [1, 2]. The core symptom is gait disturbance, which is also the most responsive to the surgical implantation of a shunt system, which reduces symptoms in up to 86% of patients [3, 4].

Idiopathic NPH primarily affects the elderly and shows a progressive increase in prevalence after 65 years [5]. Given this age distribution, it is expected that a significant percentage of patients will harbor neurodegenerative changes related to common age-related disorders such as Alzheimer disease (AD) and Lewy body disease (LBD). The latter defines a clinically heterogeneous group of α-synucleinopathies characterized by tissue deposition of misfolded alpha-synuclein (α-syn) forming Lewy bodies (LB), which includes Parkinson disease (PD), Parkinson disease dementia (PDD), and dementia with Lewy bodies (DLB).

However, the prevalence of LB- and AD-related pathologies in patients with iNPH and, most importantly, their role in clinical features, disease progression, and response to treatment remain largely unexplored. Studies in vivo based on pathology-driven cerebrospinal fluid (CSF) biomarkers have shown a variable prevalence, ranging from 17–49%, of pathological changes related to protein beta-amyloid (Aβ) deposition, which are likely dependent on patient selection criteria [6, 7]. Other studies have shown an association between phosphorylated tau (p-tau) levels or the amyloid-beta 1–42 (Aβ42)/p-tau ratio and cognitive decline. A higher p-tau also correlated with a poorer cognitive outcome after surgery [8].

In contrast to AD biomarkers, no study has explored to date the prevalence of LB pathology in iNPH, a goal that, until recently, could have only been accomplished through a neuropathologic evaluation. However, the real-time quaking-induced conversion (RT-QuIC), an ultrasensitive assay that detects misfolded α-syn in CSF using an amplification strategy, has recently provided a robust in vivo biomarker for LB pathology, even when present as co-pathology [9–11]. In this regard, we recently demonstrated that approximately 15% of patients diagnosed with mild cognitive impairment due to AD are also affected by LB pathology [12].

In addition to pathology-specific CSF biomarkers, neurofilament light chain (NfL), a nonspecific marker of axonal degeneration, has attracted increasing attention as a sensitive biomarker of disease severity and progression in many neurological disorders. Studies on NfL in iNPH are scant and have provided variable results [8, 13, 14]. However, a recent study found an association between CSF NfL levels and cognitive and gait performance in iNPH patients [7].

The main objective of our study was to determine the rate of α-syn seeding activity detected by RT-QuIC in a large iNPH sample belonging to two well-characterized prospective cohorts. Secondary aims were the investigation of 1) the possible associations between CSF biomarkers and iNPH clinical features and 2) the role of CSF biomarkers in predicting the short-term outcome after surgery.

## Methods

### Study cohorts, patient selection, and clinical protocols

To estimate the prevalence of LB-related co-pathology in iNPH (primary aim), we studied 293 patients recruited retrospectively from two prospective cohorts, the Bologna PRO-HYDRO study (n = 127) [15] and the Kuopio idiopathic normal pressure hydrocephalus cohort (n = 166) [16]. The protocols applied to the Italian and Finnish cohorts have been previously described [15, 16]. In both groups, we assigned the diagnosis of iNPH following consensus guideline criteria [2], and all 293 patients fulfilled the criteria for probable iNPH [2]. CSF samples were collected preoperatively in both groups.

Patients were selected for ventriculoperitoneal CSF shunt surgery as described [15, 16]. Given the heterogeneity of the clinical/instrumental data protocols, including the timing of data collection between the two cohorts, we investigated the associations between CSF biomarker profiles and the clinical/instrumental features before and after surgery only in the Italian cohort (secondary aims 1 and 2). In the latter, we evaluated clinical symptoms at CSF sampling and 6 months after shunt surgery. The clinical evaluation, carried out by the same team, included the following assessments: partial (section III) Movement Disorder Society-sponsored revision of the Unified Parkinson's Disease Rating Scale (MDS-UPDRS) motor score [17], postural stability and tandem walking, 18-meters walking test [15] and Timed up and go test (TUGT) [18], urinary symptoms, iNPH Grading Scale [19], Gait Status Scale [19], Tinetti Assessment Tool [20] and Modified Rankin Scale (mRankin) [21]. Moreover, all patients underwent neuropsychological testing [15]. Three independent operators reviewed magnetic resonance images blinded to the clinical data to assess vascular comorbidity using the Fazekas [22], and the Age-Related White Matter Changes (ARWMC) scales [23]. In those who underwent shunt surgery, we defined a favorable outcome as a patient showing an improvement ≥ 1 point on the mRankin scale [21].

To compare the prevalence of α-syn seeding activity between iNPH and a patient cohort that might represent the general population, we studied 89 consecutive, age-matched patients with definite or probable Creutzfeldt-Jakob disease (CJD according to current diagnostic criteria [24]). We chose this cohort because of the availability of post-mortem neuropathologic examination in most cases. Moreover, there is no evidence for an increased genetic risk for LBD in prion disease.

Finally, to comparatively evaluate the kinetic proprieties of α-syn RT-QuIC reactions (see below) between iNPH and a well-defined LBD cohort, we included 45 consecutive patients diagnosed with probable DLB according to McKeith criteria [25].

A summary of all the analyses performed in each patient group is provided in Additional file 1: Table S1.

### CSF biochemical analysis

#### Sample collection

At both the Istituto delle Scienze Neurologiche di Bologna (ISNB) and the Kuopio University hospital, CSF samples were obtained by a lumbar puncture at tap test, centrifuged routinely (in Kuopio) or in case of blood contamination (even mild) (in Bologna), divided into aliquots, and stored in polypropylene tubes at −80 °C until analysis. All analyses except the AD core markers assessment in Kuopio cohort were carried out at ISNB by personnel blinded to patients' diagnoses.

#### Alpha-synuclein real-time quaking-induced assay

We performed the CSF α-syn RT-QuIC assay, including purification of recombinant wild-type human α-syn, as previously described [10, 12]. Briefly, we ran the same positive and negative control samples throughout all experiments to optimize the comparison between fluorescent responses in different plates. To overcome batch-to-batch variations and intrinsic plate-to-plate variability, we normalized the relative fluorescent units (RFU) for every time point to the median of the maximum intensity reached by four positive control replicates within each plate and expressed it as a percentage. We then set the threshold at 20% of the abovementioned parameter and the cut-off at 30 h. When only one of the four replicates crossed the threshold, the analysis was considered "unclear" and repeated up to three times. In those participants who showed a positive RT-QuIC α-syn seeding profile (at least 2 out of 4 positive replicates), we measured the peak of the fluorescence response (Imax) and the lag phase (LAG) (time required to reach the threshold).

#### Alzheimer's disease core biomarkers and neurofilament light chain

In CSF samples from the Bologna PRO-HYDRO study, we measured total tau (t-tau), p-tau, Aβ42, and amyloid-beta 1–40 (Aβ40) by automated chemiluminescent enzyme-immunoassay (CLEIA) on the Lumipulse G600II platform (Fujirebio Europe NV, Gent, Belgium). The mean interassay coefficients of variation (CVs) were ˂ 8% for all biomarkers. We calculated the Aβ42/40 ratio according to a previously published formula [26]. In the same CSF samples, we measured NfL concentration by a validated commercial enzyme-linked immunosorbent assay (ELISA) (NfL ELISA kit, IBL, Hamburg, Germany) [27]. The mean intra- and interassay CVs for NfL analyses were 2 and 10%, respectively. In the Finnish cohort, the CSF levels of Aβ42, t-tau, and p-tau were measured by commercial ELISA kits (Innotest β-amyloid1–42, Innotest Tau-Ag, Innotest Phosphotau (181P), Fujirebio, Ghent, Belgium) using the manufacturer's protocol. The cut-off values for Aβ + status were as follows: Aβ42/40 ratio < 0.65 in the Bologna cohort [12] and Aβ42 < 500 mg/mL in the Kuopio cohort [28].

### Statistical analysis

The normality of continuous parameter distribution was checked using the skewness-kurtosis test, and variables were expressed as the mean ± standard deviation (SD) or median along with interquartile ranges (IQRs) when appropriate. Continuous variables were compared by using the t-test or Wilcoxon rank-sum test, as appropriate. Categorical variables were described by their absolute and/or relative frequencies and compared using the chi-square test.

A logistic regression model was used to calculate the odds ratio (OR) and 95% confidence interval (CI) to assess the association between surgery outcome (dependent variable) and CSF biomarkers. Adjustment for age, sex and scales (mRankin scale, gait impairment, MDS-UPDRS motor score) at baseline was performed through a multivariable-adjusted logistic regression analysis. After a sensitivity analysis, the final multivariable model was revised, including just the most important predictors and excluding highly correlated features. Spearman correlations were performed to assess correlations among variables.

A p-value lower than 0.05 (2-sided) was considered significant. Statistical analyses were performed using the statistical software STATA^®^, version 14.0.

## Results

The demographic and clinical characteristics of the iNPH cohorts are summarized in Table 1.

**Table 1:** Baseline clinical features of the total iNPH sample

	Total iNPH samples	Bologna cohort	Kuopio cohort	p-value
	293	127	166	
Males, *n (%)*	171 (58.4)	83 (65.4)	88 (53.0)	0.034
Age at evaluation, *years*	75.4 ± 5.7	75.7 ± 5.1	75.1 ± 6.5	0.53
BMI, *(kg/m*^*2*^*)*	27.0 (24.5–29.3)	26.8 (24.2–29.6)	27.0 (24.7–29.1)	0.86
Age at disease onset, *years*	73.0 (69.0–77.0)	73.0 (69.0–76.0)	72.9 (68.7–78.3)	0.5
Disease duration < 12 months, *n (%)*	53 (18.5)	21 (16.5)	32 (19.3)	0.68
**Symptoms at first evaluation**				
Gait disorders, *n (%)*	285 (97.3)	119 (98.4)	166 (100)	0.18
Urinary dysfunctions, *n (%)*	243 (82.9)	100 (78.7)	143 (86.1)	0.42
Cognitive impairment, *n (%)*	220 (75.1)	83 (65.4)	137 (82.5)	0.004
**Urinary dysfunctions**				
Urinary incontinence, *n (%)*	159 (54.3)	74 (58.3)	85 (51.2)	0.037
Urinary urgency, *n (%)*	241 (82.3)	98 (77.2)	143 (86.1)	0.05
Gait Speed *(m/s)*	0.69 ± 0.31	0.70 ± 0.27	0.67 ± 0.34	0.25
**Scores**				
iNPH grading scale (max 12)	6 (4–8)	6 (4–7)	7 (4–9)	0.01
mRankin Scale (max 6)	2 (2–3)	2 (1–3)	3 (2–3)	0.001
MMSEc (max 30)	25 (21–27)	26 (24–28)	23 (20–26)	0.001
Dopaminergic treatment				
Levodopa treatment, *n (%)*	40 (13.7)	36 (28.4)	4 (2.4)	< 0.001
Levodopa max daily posology *(mg)*	300 (300–450)	300 (300–450)	450 (200–800)	0.64
Other antiparkinsonian drugs, *n (%)*	10 (3.4)	6 (4.7)	4 (2.4)	0.28
Shunt Surgery, *n (%)*	255 (87.0)	89 (70.1)	166 (100)	< 0.001
**Biomarkers**				
α-syn RT-QuIC + , *n (%)*	60 (20.5)	28 (22.1)	32 (19.1)	0.56
t-tau (pg/ml)	184 (137–252)	189 (142–256)	182 (129–240)	0.13
p-tau (pg/ml)	28 (20–40)	26 (21–35)	30 (17–43)	0.82
NfL (pg/ml)	–	1018 (755–1483)	–	–
Aβ42 (pg/ml)	573 (424–777)	495 (365–714)	662 (513–856)	< 0.001
Aβ42/40 ratio	–	0.84 ± 0.21	–	–
Aß + ^§^, *n (%)*	60 (24.0)	32 (25.2)	28 (22.4)	0.58

Continuous variables are expressed as the mean ± SD or median (IQR)

*Aβ40* amyloid-beta 1–40, *Aβ42* amyloid-beta 1–42, *BMI* Body Mass Index, *m* meters, *MMSEc* corrected Mini-Mental State Examination, *mRankin* Modified Rankin Scale, *NfL* neurofilament light chain protein, *iNPH* idiopathic normal pressure hydrocephalus, *p-tau* phosphorylated tau protein, *s* seconds, *t-tau* total tau protein, *RT-QuIC* real-time quaking-induced conversion

^§^Aß cut-offs: Bologna cohort, Aβ42/40 ratio < 0.65; Kuopio cohort, Aβ42 < 500 pg/ml

There were significant differences in sex, clinical features, and scale scores between the two cohorts. The percentage of males was higher in the Bologna cohort (65.4% vs. 53.0%, p = 0.034). In contrast, patients in the Finnish cohort showed a higher rate of cognitive impairment at the first evaluation (82.5% vs. 65.4%, p = 0.004), higher scores on the iNPH grading scale [7 (4–9) vs. 6 (4–7), p = 0.01] and mRankin [3 (2–3) vs. 2 (1–3), p = 0.001] scales, and lower corrected Mini-Mental State Examination (MMSEc) scores [23 (20–26) vs. 26 (24–28), p = 0.001] (Table 1). We also found divergent Aβ42 values between cohorts that likely reflected the different methodologies used for biomarker analyses (automated CLEIA vs. manual ELISA).

Detailed information about the CJD and DLB cohorts is provided in Additional file 1: Table S2 and Table S3).

### Prevalence and characteristics of α-synuclein seeding activity in patients with iNPH from the two cohorts

Sixty out of 293 (20.5%) iNPH patients showed α-syn seeding activity by the RT-QuIC assay, with a similar prevalence between the Italian (22.1%) and Finnish (19.3%) cohorts (p = 0.563). Notably, the percentage of α-syn-positive patients in the iNPH group significantly exceeded that of the age-matched CJD cohort, in which only 6 out of 89 (6.7%) patients showed positive RT-QuIC reactions (p = 0.002) (Additional file 1: Table S2).

**Fig. 1:**
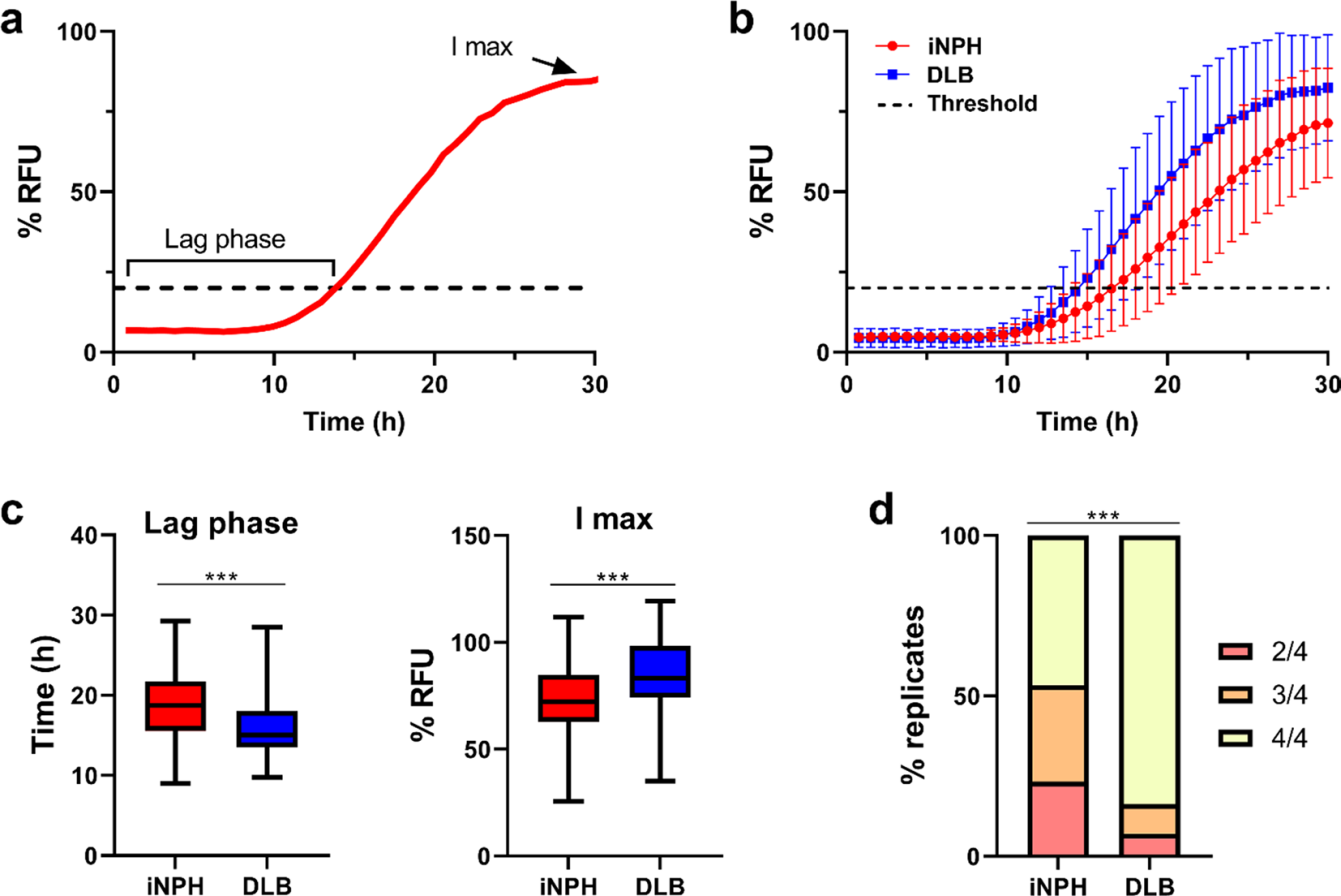
α-syn RT-QuIC kinetic parameters in the study cohort. **a** Representation of the analyzed kinetic parameters. The Lag phase represents the time interval between the beginning of the reaction and the time in which the fluorescent signal crosses the threshold (dashed line); the I max is the maximum fluorescence value reached by the curve. **b** Differences in the mean normalized fluorescence emission of α-syn RT-QuIC positive cases between iNPH (red line) and DLB (blue line) clinical cases. The black dashed line represents the threshold. The error bars indicate the standard deviation (SD). **c** The comparison of kinetic parameters of α-syn RT-QuIC positive cases between the two groups (iNPH and DLB) shows statistically significant differences in lag phase and I max (***p ≤ 0.001). **d** Distribution analysis of positive replicates in the iNPH and DLB cohorts. In **b**, **c**, and **d**: iNPH, n = 60 and DLB, n = 45. Statistical analysis was performed by Chi-square test (***p ≤ 0.001)

The comparison of the RT-QuIC kinetic parameters between the iNPH and DLB groups showed reduced kinetics of the seeding activity (i.e., more extended lag phase and lower Imax) in the former group (lag phase: iNPH 18.8 h vs. DLB 16.1 h, p < 0.001; Imax: iNPH 73.3% vs. DLB 85.4%, p < 0.001) (Fig. 1). In line with the latter observation, we found a higher percentage of cases showing a complete 4 of 4 positive responses in DLB patients than in those with iNPH (83.7% vs. 47.5%, p < 0.001) (Fig. 1). Accordingly, the percentages of 3/4 and 2/4 positive replicates were significantly higher in the latter group (9.3% vs. 30.0%; 7.0% vs. 25.3%). There were no significant differences between the two iNPH cohorts in the percentage of α-syn RT-QuIC-positive (α-syn^LB^ +) patients showing 4/4, 3/4, and 2/4 positive replicates (Italian cohort vs. Finnish cohort; 4/4: 39.3% vs. 53.1%, 3: 32.1% vs. 28.1% and 2/4: 28.6% vs. 18.8%, p = 0.519).

### Association between α-syn^LB^ + and clinical variables in the Italian cohort

**Fig. 2:**
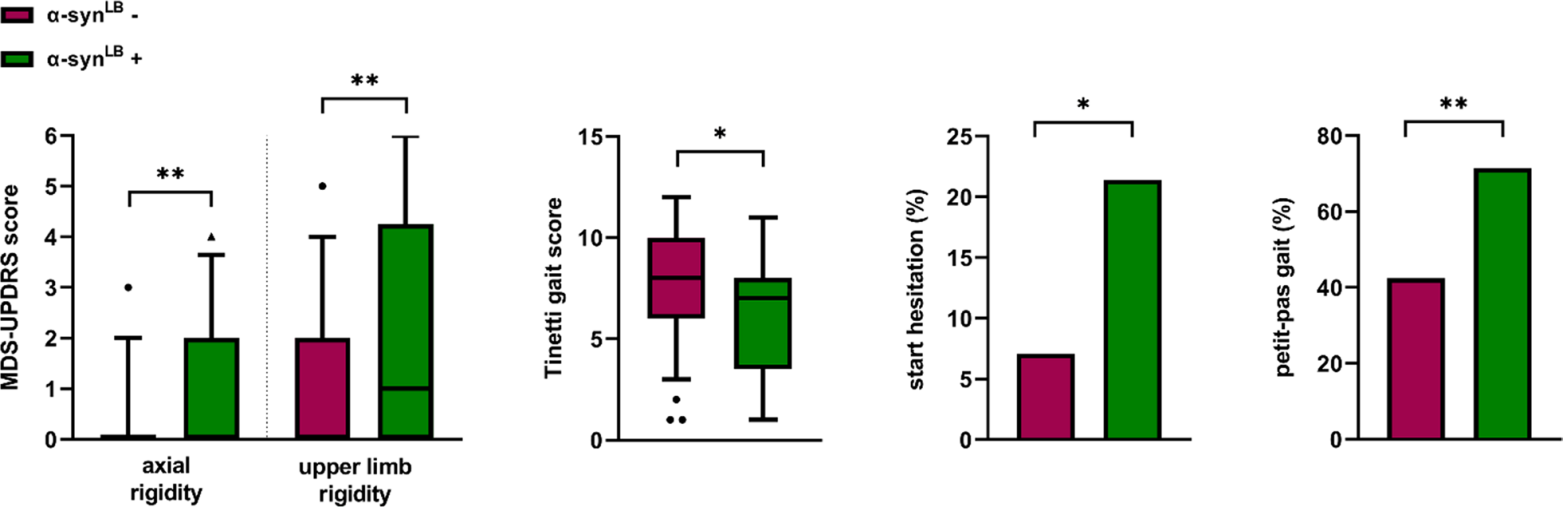
Differences in motor performance after stratifying individuals according to α-syn RT-QuIC results. Data from the Bologna PRO-HYDRO cohort (α-syn^LB^ +, n = 28 and α-syn^LB^ -, n = 99). *p ≤ 0.05, **p ≤ 0.01. Legend = α-syn^LB^: Lewy body-associated α-synuclein seeding activity MDS-UPDRS: Movement Disorder Society-Unified Parkinson's Disease Rating Scale

Comparisons between the α-syn^LB^ + and α-syn^LB^ – groups were performed only in the Italian cohort (Additional file 1: Table S1). There were no differences in demographic and clinical features, including urinary symptoms, at disease onset. However, the α-syn^LB^ + group scored higher on axial and upper limb rigidity than the α-syn^LB^ – group on neurological examination (Fig. 2).

There was also a trend toward a higher partial MDS-UPDRS motor score in the α-syn^LB^ + group, mainly related to the higher score on upper limb rigidity and bradykinesia items. Patients with α-syn seeding activity also showed a trend toward a higher prevalence of resting tremor (25.0% vs. 16.2%) (Additional file 1: Table S4). Concerning gait assessment, the α-syn^LB^ + group more frequently showed a petit-pas gait (71.4% vs. 42.4%, p = 0.007), start hesitation (21.4% vs 7.1%, p = 0.036) (Fig. 2), and despite not statistically significant, arm swing reduction (64.3% vs. 52.5%) and freezing of gait (21.4% vs 9.1%) compared to the α-syn^LB^ - group. They also showed a reduced gait speed [0.6 (0.5–0.8) vs. 0.8 (0.5–0.9) meters per second], but the difference did not reach statistical significance (Additional file 1: Table S4).

**Fig. 3:**
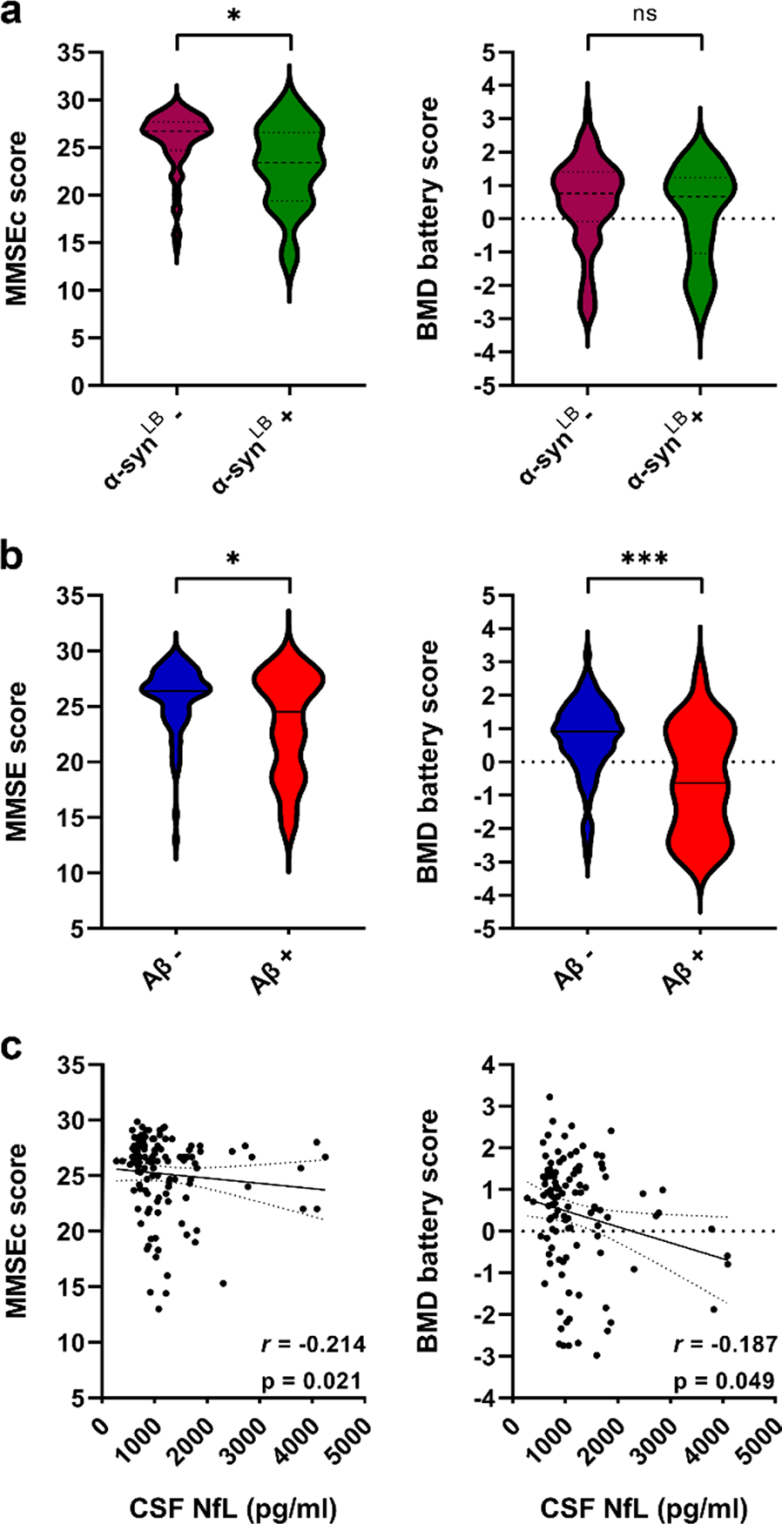
Comparisons of neuropsychological test results according to α-syn^LB^ status (α-syn^LB^ +, n = 28 and α-syn^LB^ -, n = 99) **a** and Aβ status (Aβ+, n = 32 and Aβ-, n = 94) **b** and correlations with NfL levels **c**. α-syn^LB^ status according to RT-QuIC results (positive: + , negative: -). Aβ status according to the Aβ42/40 ratio < 0.65 (Aβ +), > 0.65 (Aβ-). *p ≤ 0.05, ***p ≤ 0.001. Legend = α-syn^LB^: Lewy body-associated α-synuclein seeding activity; MMSEc: corrected Mini-Mental State Examination; BMD: Brief Mental Deterioration; ns: not significant

iNPH patients who tested positive by RT-QuIC also showed an increased fall risk and higher gait impairment measured by the Tinetti gait section [7 (4–8) vs. 8 (6–10), p = 0.017] and scored worse on the MMSEc [23.4 (19.4–26.3) vs. 26.7 (24.7–27.7), p = 0.003] (Fig. 3). Finally, patients in the α-syn^LB^ + group more frequently consumed levodopa (42.9% vs. 24.2%) and revealed a levodopa response more frequently (25.0% vs. 8.1%), despite the similar posology between groups. These results, although clinically relevant, did not reach statistical significance (Additional file 1: Table S4).

### Association between Aβ + or NfL levels and clinical variables in the Italian cohort

In the Bologna cohort, Aβ + patients (n = 32, 25.4%) showed fewer years of education [8 (5–8) vs. 11 (5–13), p = 0.014] and lower scores on the MMSEc [24.5 (19.3–27.4) vs. 26.4 (24.7–27.7), p = 0.037] and Brief Mental Deterioration Battery [−0.63 (−2.18–0.84) vs. 0.91 (0.28–1.47), p = 0.0003] than Aβ – (Fig. 3, Additional file 1: Table S5). Moreover, the Aβ + group more frequently showed cognitive impairment, both as first symptoms at disease onset (21.9% vs. 8.5%) and during the disease course (71.9% vs. 62.8%), although these results did not reach statistical significance.

CSF NfL levels showed a statistically significant association with the following categorical variables: sex (p = 0.018), cognitive impairment (p = 0.029), resting tremor (p = 0.027), and levodopa intake (p = 0.035). Continuous variable significantly correlating with NfL values included age (rho = 0.224, p = 0.011) and MMSEc (rho = -0.213; p = 0.021) (Fig. 3).

### Outcome after shunt surgery in the Italian cohort

**Fig. 4:**
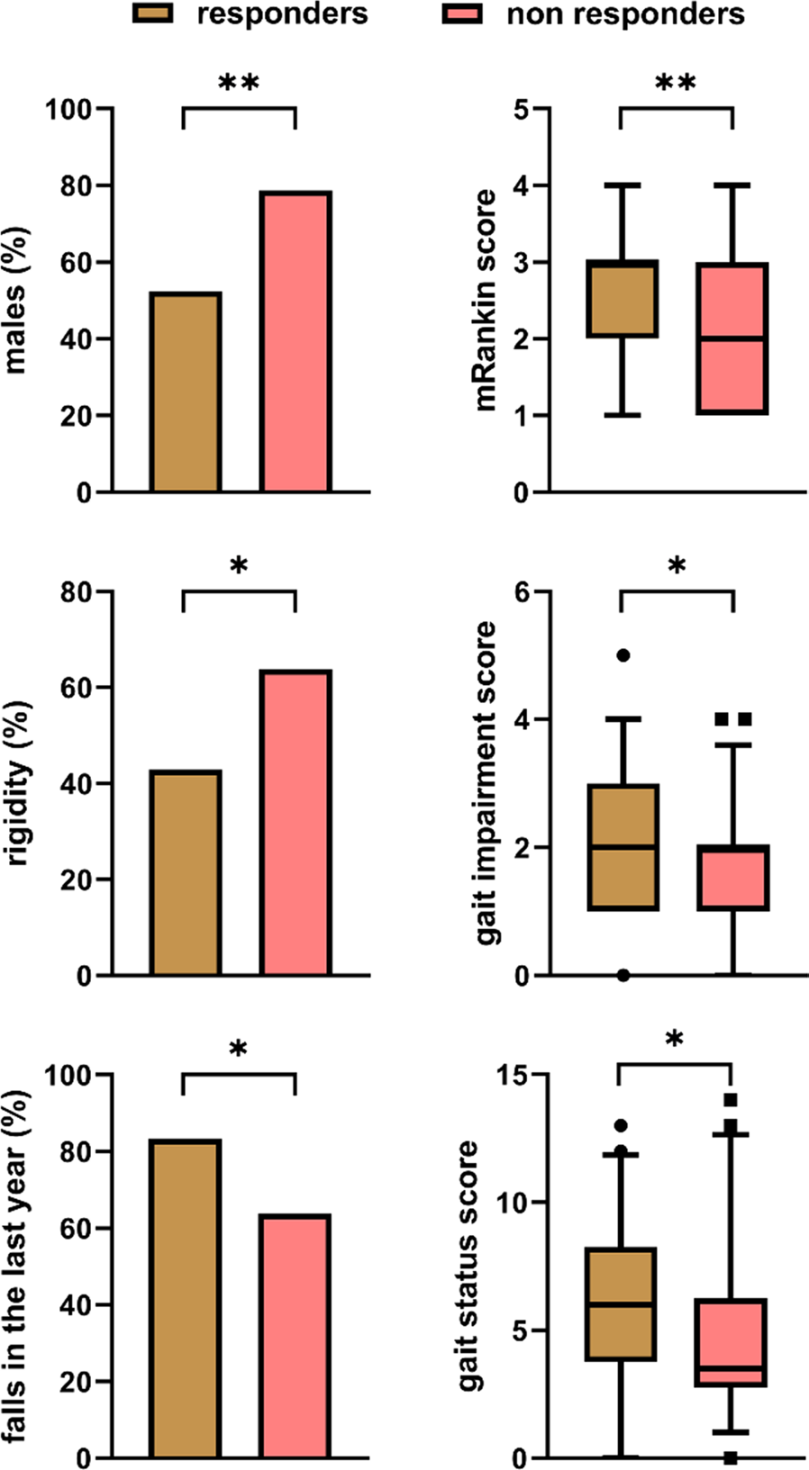
Discrepant clinical features at baseline in patients classified as responders (n = 42) vs. non-responders (n = 47) at 6 months after shunt surgery. Data from the Bologna PRO-HYDRO cohort. *p ≤ 0.05, **p ≤ 0.01

Eighty-nine patients in the Bologna cohort were evaluated 6 months after ventriculoperitoneal shunt surgery; 42 were classified as responders, and 47 as non-responders. Responders were predominantly females (47.6% vs. 21.3%, p = 0.009) and presented more severe symptoms at baseline than non-responders. These included higher gait impairment [2 (1–3) vs. 2 (1–2), p = 0.035], higher rate of fall (83.3% vs. 63.8%, p = 0.038), and worse scores on the Gait Status [6 (4–8) vs. 3.5 (3–6), p = 0.025] and mRankin scales [3 (2–3) vs. 2 (1–3), p = 0.008) (Fig. 4, Additional file 1: Table S6). In contrast, non-responders manifested rigidity more frequently (63.8% vs. 42.9%, p = 0.035). They also more often showed cognitive impairment, both as the first symptom at disease onset (23.4% vs. 7.1%) and at first evaluation (76.6% vs. 59.5%) and levodopa intake (40.4% vs. 21.4%); however, these differences did not reach statistical significance. No statistically significant differences in CSF biomarker values emerged between the two groups.

In the univariate analysis, the following clinical variables were significantly associated with outcome (responders vs. non-responders): sex [females vs. males (reference values), OR  3.36, 95% CI  1.33–8.48), baseline mRankin (OR   1.79, 95% CI 1.14–2.81), baseline gait impairment (OR 1.62, 95% CI 1.01–2.50), baseline Gait Status Scale score (OR 1.14, 95% CI 1.01–1.29) and treatment with levodopa (OR 0.40, 95% CI 0.16–1.03). In the univariate analysis, neither the continuous CSF biomarker levels nor positivity for α-syn RT-QuIC was associated with outcome after surgery.

Gait impairment, the Gait Status Scale score, and the mRankin score were strongly correlated (p < 0.0001).

After exclusion of highly correlated variables, the best multivariable model retained the following significant variables (adjusted for age): sex (females vs. males, OR 3.28, 95% CI 0.89–1.09), mRankin scale at baseline (OR 2.21, 95% CI 1.25–3.90) and levodopa treatment (OR 0.26, 95% CI 0.08–0.85).

## Discussion

To determine the prevalence of LB pathology in iNPH, we applied the CSF α-syn RT-QuIC assay to a large group of 293 patients belonging to two well-characterized cohorts. We found that approximately 20% of patients with iNPH harbor α-syn seeding activity related to LB pathology. In contrast, α-syn RT-QuIC showed a positive reaction in only 6.7% of the age-matched CJD cohort we used as a control group. Previous studies showing a 9 to 13% rate of incidental LB pathology in cognitively and neurologically normal elderly subjects also support our conclusion of a significant association between LBD and iNPH [29–32]. This finding indicates a causal link between LB pathology and clinical symptoms and disease progression in a subgroup of iNPH patients. In support of this conclusion, patients who tested positive by α-syn RT-QuIC had a higher score on axial and upper limb rigidity and showed a more significant gait impairment characterized by petit-pas gait and start hesitation. Additionally, the α-syn^LB^ + group showed arm swing reduction, freezing of gait, and a reduced gait speed. They also consumed levodopa more frequently.

Altogether, these neurological signs define a predominant parkinsonian phenotype of iNPH, which the LB pathology might at least partially sustain. Indeed, parkinsonian features, including short and symmetrical steps, freezing of gait, start hesitation, magnetic gait, bradykinesia, and rigidity, belong to the clinical spectrum of iNPH [33]. Of note, none of the patients included in our study fulfilled the criteria for a clinical diagnosis of probable or clinically established PD, PDD, or DLB. Indeed, the finding of α-syn seeding activity by RT-QuIC in patients fulfilling the diagnostic criteria for both iNPH and LBD would be expected, as shown in a recent study [34]. In this context, the results of our study suggest the existence of a spectrum of LB pathology in patients with iNPH variably contributing to clinical features, especially parkinsonian signs. We also demonstrated that α-syn RT-QuIC is a valuable biomarker for identifying these patients. Concerning dopaminergic treatment, patients with iNPH usually respond poorly to levodopa [35]. In our sample, the α-syn^LB^ + group more frequently consumed levodopa, suggesting a subjective clinical benefit, and more frequently showed a levodopa response. Therefore, α-syn RT-QuIC might also help to identify patients who might benefit from therapy with levodopa.

As an indication of a disturbance in the nigrostriatal pathway, two studies found striatal dopaminergic deficits on dopamine transporter scans in 31% and 47% of iNPH patients [36, 37]. In a more recent study, dopamine transporter binding reduction mainly affected the subgroup of iNPH patients with more prominent gait involvement and higher MDS-UPDRS III scores [38]. Future studies should investigate α-syn seeding activity by RT-QuIC in these patients to verify the association between RT-QuIC positivity and the nigrostriatal dopaminergic deficit assessed in vivo.

In our study, Aβ + patients showed lower scores on MMSEc and Brief Mental Deterioration Battery evaluations than Aβ- patients. This result confirms previous findings showing an association between lower Aβ42 levels and cognitive impairment [7] due to AD comorbidity, as also reported in neuropathological studies [39, 40]. The AD pathology comorbidity could lead to a specific iNPH phenotype with predominant cognitive domain involvement. According to our results, this subgroup of patients more frequently present with cognitive impairment at disease onset, which is important for the differential diagnosis at an early disease stage and patient management.

Our data showing an association between CSF NfL levels and reduced cognitive performance partially aligns with previous studies showing a correlation between NfL levels and the severity of clinical symptoms in iNPH patients [7, 8, 13, 14, 41]. In recent cohorts of 65 Finnish and 82 Swedish iNPH patients, NfL was also negatively correlated with preoperative gait velocity [8]. Finally, higher NfL was associated with lower scores on the total iNPH scale and motor, balance, and continence domains in a large cohort of 455 patients [7]. The lack of a positive correlation with symptom severity in motor and urinary domains in our cohort may reflect heterogeneity in selection criteria. For example, we paid significant attention to excluding patients with substantial vascular burden in subcortical white matter, notoriously affecting NfL levels and motor performance.

Concerning surgery outcome, our results suggest that currently available CSF biomarkers for LB and Aβ pathologies and myelinated axon degeneration do not significantly predict the short-term (6 months) response after a shunt. Therefore, despite the clinical variability of iNPH, correlating with the biomarker profile, iNPH patients with some degree of neurodegenerative copathology could also benefit from surgery, improving both clinical symptoms and functional independence.

Previous studies investigated the association between CSF Aβ42, tau, or NfL and outcome after surgery. In line with our results, these studies, including a recent meta-analysis of four studies with a pooled sample size of 254 shunted patients, found that preoperative Aβ42 did not correlate with the shunt response [7, 8, 13, 27, 42–44]. In the Italian cohort, we also measured the Aβ42/Aβ40 ratio, which reflects Aβ brain pathology more accurately than Aβ42 alone, and confirmed the lack of association with the clinical outcome after surgery.

The abovementioned meta-analysis, which included four studies with a pooled sample size of 254 shunted patients for CSF p-tau analysis and six studies with a pooled sample size of 310 shunted patients for CSF t-tau, indicated higher levels of CSF p-tau and t-tau in shunt-nonresponsive than in shunt-responsive iNPH subjects [44]. Recently, two studies conducted on a sizeable ìNPH sample and not included in the meta-analysis found an association between higher levels of t-tau and reduced improvement after shunt surgery in a cohort of 455 patients [7] and a negative correlation between p-tau and postoperative outcome in a cohort of 82 patients [8]. In our cohort of 89 Italian patients who underwent surgery, the percentage of those with elevated t-tau or p-tau levels was meager, limiting the power of statistical analysis. This result likely reflects the recruitment strategy in our center, which is more focused on movement disorders than cognitive decline.

Correlations between CSF NfL levels and surgery outcomes remain controversial. Preoperative higher levels of NfL were associated with a poor outcome in a recent study on a Swedish cohort of 455 patients [7], while other studies, including the present one, did not confirm the association [8, 13, 14, 41].

The discrepancy in results among studies evaluating the outcome after surgery could be related to the heterogeneity in study design, sample size, and method of CSF biomarker assessment. Moreover, the lack of shared and recognized guidelines, the heterogeneity in outcome measures, and the time of outcome evaluations likely negatively affect the comparison between studies. For example, outcome measures utilized to date included subjective improvement, improvement of at least 1 point on gait score, of 5% or 20% in gait performances, of 1–3 points in the iNPH grading scale [19], of > 5 points in the iNPH scale [45], or in mRankin score [21]. Similarly, the postsurgery evaluation included different time-lapses (i.e., 3, 6, and 12 months postoperatively). In our study, responders were defined based on 1 point in mRankin to identify patients with an improvement impacting dependence on activities of daily living and disability. However, for a better comparison with previous studies, we also considered responders patients with a decrease of at least 1 point on the iNPH grading scale. Still, the analysis confirmed the lack of an association between the CSF biomarkers examined and the surgical outcome at 6 months.

The strengths of our study are the inclusion of two well-characterized cohorts and the systematic method of analysis applied to the Italian cohort, including several clinical parameters, 3-Tesla-MRI with vascular burden evaluation, and a comprehensive neuropsychological assessment.

The study's main limitation is the heterogeneity of clinical variables and time of data collection between the two cohorts, which has prevented aggregate analysis in the study of the association between CSF biomarker profiles and clinical data or surgery outcome.

Further analyses on larger samples and probably with a multicenter nature are required to confirm the association between biomarker profiles and clinical/instrumental data and to evaluate the predictive role of these biomarkers on long-term follow-up. Postmortem neuropathological studies should also be carried out to confirm the presence and determine the burden of LB pathology in patients with iNPH.

In conclusion, we provided evidence that LB pathology affects a subgroup of iNPH patients, contributing to a specific iNPH clinical phenotype with enriched parkinsonian features. We also presented confirmatory evidence that markers of AD pathology and nonspecific neurodegeneration are associated with more severe cognitive decline and overall symptom severity in these patients. Finally, we documented a lack of correlation between CSF marker values and short-term (6 months) shunt responsiveness. However, despite this negative association, it will be essential to investigate whether the neurodegenerative-associated copathology revealed by CSF biomarkers could affect the long-term prognosis by reducing the long-term benefit in some patients.

## Supplementary Information

**Additional file 1: Table S1.** Summary of evaluations in the included cohorts. **Table S2.** Demographic data and summary of α-syn RT-QuIC results in the CJD cohort. **Table S3.** Summary of demographic and clinical features and results of indicative biomarkers of DLB individuals. **Table S4.** Baseline clinical features of the Bologna PRO-HYDRO cohort and comparisons between α-synLB + and α-syn-LB -. **Table S5.** Comparisons between Aß + vs. Aß– subgroups of patients from the Bologna PRO-HYDRO cohort. **Table S6.** Comparisons between responders and non-responders 6 months after surgery.

## Data Availability

The datasets used and/or analysed during the current study are available from the corresponding author on reasonable request.

## Abbreviations

18-m: 18-Meters
α-syn: α-Synuclein
α-syn^LB^: Lewy body-associated α-syn
Aβ: Protein beta-amyloid
Aβ40: Beta-amyloid 1–40
Aβ42: Beta-amyloid 1–42
AD: Alzheimer disease
ARWMC: Age-related white matter changes scale
CI: Confidence interval
CJD: Creutzfeldt-Jakob disease
CLEIA: Chemiluminescent enzyme-immunoassay
CSF: Cerebrospinal fluid
CVs: Coefficient of variations
ELISA: Enzyme-linked immunosorbent assay
DLB: Dementia with Lewy bodies
iNPH: Idiopathic normal pressure hydrocephalus
IQR: Interquartile ranges
ISNB: Istituto delle Science Neurologiche di Bologna
LAG: Lag phase
LB: Lewy body
LBD: Lewy body disease
MDS-UPDRS: Movement disorder society-sponsored revision of the unified Parkinson's disease rating scale
MMSEc: Corrected mini-mental state examination
mRankin: Modified rankin scale
NfL: Neurofilament light chain
OR: Odds ratio
p-tau: Phosphorylated tau
NP-Lab: Neuropathology laboratory
PD: Parkinson disease
PDD: Parkinson disease dementia
RFU: Relative fluorescent units
RT-QuIC: Real-time quaking-induced conversion
SD: Standard deviation
TUGT: Timed up and go test
t-tau: Total tau
