# Supplementary material for: In vivo assessment of Lewy body and beta-amyloid copathologies in idiopathic normal pressure hydrocephalus: prevalence and associations with clinical features and surgery outcome
Source: Fluids Barriers CNS. 2022 Sep 7;19:71. doi: 10.1186/s12987-022-00368-2 (PMC9454182; doi:10.1186/s12987-022-00368-2)
Supplement: Supplementary file 1 — Additional file 1: Table S1. Summary of evaluations in the included cohorts. Table S2. Demographic data and summary of α-syn RT-QuIC results in the CJD cohort. Table S3. Summary of demographic and clinical features and results of indicative biomarkers of DLB individuals. Table S4. Baseline clinical features of the Bologna PRO-HYDRO cohort and comparisons between α-synLB + and α-syn-LB -. Table S5. Comparisons between Aß + vs. Aß– subgroups of patients from the Bologna PRO-HYDRO cohort. Table S6. Comparisons between responders and non-responders 6 months after surgery. [file 12987_2022_368_MOESM1_ESM.pdf]

ADDITIONAL FILE 1

SUPPLEMENTARY MATERIALS

List of contents

Table S1 2

Table S2 3

Table S3 4

Table S4 5

Table S5 7

Table S6 8

Table S1. Summary of evaluations in the included cohorts.

	**NPH Pro-Hydro**	**NPH Kuopio**	**CJD**	**DLB**
Demographic	√	√	√	√
CSF α-syn RT-QuIC	√	√	√	√
CSF Aβ	√	√		
CSF NfL	√			
Association CSF biomarkers and clinical variables	√			
Outcome evaluation	√			
Neuropathology			√	

Legend=: NPH: normal pressure hydrocephalus; CJD, Creutzfeldt-Jakob disease; DLB, dementia with Lewy bodies; RT-QuIC, real-time quaking-induced conversion; α-syn, α-synuclein; Aβ, amyloid-beta; NfL, neurofilament light chain.

Table S2. Demographic data and summary of α-syn RT-QuIC results in the CJD cohort.

	**CJD group**
N	89
Female, n (%)	46 (51.7)
Age at lumbar puncture (years)	75.2±4.9
Definite/probable*, n (%)	60/29 (67.4/32.6)
Positive α-syn RT-QuIC, n (%)	
- Definite	4 (6.7)
- Probable	2 (6.9)

*Definite and probable CJD diagnoses were formulated according with the updated European diagnostic criteria (https://www.eurocjd.ed.ac.uk/node/833).

Legend= CJD: Creutzfeldt-Jakob disease; RT-QuIC: real-time quaking-induced conversion; α-syn: α-synuclein.

Table S3. Summary of demographic and clinical features and results of indicative biomarkers of DLB individuals.

	**DLB group**
N	45
Female, n (%)	17 (37.8)
Age at lumbar puncture (years)	73.7±5.9
Time between disease onset and lumbar puncture (years)	5.4±4.6
Clinical features, n (%)	
- Dementia (*essential feature*)	45 (100)
- Visual hallucinations	31 (68.9)
- Fluctuations	17 (37.8)
- Parkinsonism	40 (88.9)
- REM sleep behavior disorder	20 (44.4)
Indicative biomarkers, positive/tested (%)	
- SPECT DaTscan	20/22 (90.9)
- ^{123}I-MIBG myocardial uptake	13/13 (100)
- REM sleep without atonia at polysomnography	7/14 (50.0)

Legend= DLB: dementia with Lewy bodies; MIBG: metaiodobenzylguanidine.

Table S4. Baseline clinical features of the Bologna PRO-HYDRO cohort and comparisons between α-syn^{LB} + and α-syn^{LB} -.

	**Bologna iNPH cohort**	**α-syn^{LB} +**	**α-syn^{LB} -**	***p-value***
**N**	127	28	99	
**Males**, *n (%)*	83 (65.4)	16 (57.1)	67 (67.7)	0.208
**Age at evaluation**, *years*	76.0 (72.0-79.0)	76.0 (72.0-80.5)	76.0 (72.0-79.0)	0.760
**Age at disease onset**, *years*	73.0 (69.0-76.0)	74.5 (69.0-78.0)	72.0 (69.0-76.0)	0.313
**Disease duration <1 year**, *n (%)*	21 (16.5)	6 (21.4)	15 (15.2)	0.447
**Symptoms at first evaluation**				
Gait disorders, *n (%)*	119 (98.4)	25 (89.3)	94 (94.9)	0.385
Urinary dysfunctions, *n (%)*	100 (82.6)	23 (82.1)	77 (77.8)	0.560
Cognitive impairment, *n (%)*	83 (68.6)	20 (71.4)	63 (63.6)	0.349
**Symptom of disease onset**				
Gait disorders, *n (%)*	77 (64.2)	17 (60.7)	60 (60.6)	0.581
Balance disorders, *n (%)*	14 (11.7)	1 (3.6)	13 (13.1)	
Urinary dysfunctions, *n (%)*	14 (11.7)	4 (14.3)	10 (10.1)	
Cognitive impairment, *n (%)*	15 (12.5)	3 (10.7)	12 (12.1)	
**Fall in the last year**, *n (%)*	88 (72.7)	20 (71.4)	68 (68.7)	0.804
**Urinary dysfunctions***				
**Neurological examination**				
Partial MDS-UPDRS, motor section score	6.0 (3.0-14.0)	10.0 (4.0-18.0)	6.0 (3.0-12.5)	0.164
Rest tremor, *n (%)*	23 (18.1)	7 (25.0)	16 (16.2)	0.262
Gait impairment, *n (%)*	2 (1-2)	2 (1-2)	2 (1-2)	0.302
Festination, *n (%)*	15 (12.4)	3 (10.7)	12 (12.1)	0.592
Heel height reduction, *n (%)*	89 (70.1)	20 (71.4)	69 (69.7)	0.443
Petit-pas gait, *n (%)*	62 (52.1)	20 (71.4)	42 (42.4)	**0.007**
Varus, *n (%)*	59 (46.5)	9 (32.1)	50 (50.5)	0.066
Wide-based gait, *n (%)*	78 (61.4)	18 (64.3)	60 (60.6)	0.326
Arm swing reduction, *n (%)*	70 (59.3)	18 (64.3)	52 (52.5)	0.174
Freezing, *n (%)*	15 (11.8)	6 (21.4)	9 (9.1)	0.074
Start hesitation, *n (%)*	13 (10.2)	6 (21.4)	7 (7.1)	**0.036**
**TUGT** *(seconds)*	4.7 (3.7-13.3)	4.8 (4.0-20.0)	4.7 (3.7-12-7)	0.367
**18-meters walking test**				
Time 18-m *(seconds)*	25.5 (19.8-33.0)	29.4 (20.9-33.4)	23.9 (19.5-32.5)	0.212
Gait Speed *(meters for second)*	0.7 (0.5-0.9)	0.6 (0.5-0.8)	0.8 (0.5-0.9)	0.123
Step length *(meters)*	1.4 (0.5-2.1)	1.5 (0.4-2.0)	1.3 (0.5-2.2)	0.636
**Number of steps (180° turn)**	4 (3-6)	5 (4-6)	4 (3-6)	0.414
**Postural Stability**	2 (1-3)	2 (1-3)	2 (2-3)	0.289
**Scores**				
iNPH grading scale (max 12)	6 (4-7)	6 (5-7)	5 (4-7)	0.330
Gait status scale (max 16)	5 (3-8)	5 (3-9)	5 (2-8)	0.172
Tinetti balance	13 (10-15)	12 (10-14)	13 (10-15)	0.222
Tinetti gait	8 (5-9)	7 (4-8)	8 (6-10)	**0.017**
Tinetti total score (max 28)	21 (15-24)	19 (14-21)	22 (16-25)	0.052
FIM scale (max 126)	112 (100-121)	107 (98-117)	114 (100.5-121.5)	0.225
mRankin Scale (max 6)	2 (1-3)	3 (2-3)	2 (1-3)	0.075
MMSEc (max 30)	26.3 (23.7-27.7)	23.4 (19.4-26.3)	26.7 (24.7-27.7)	**0.003**
Brief Mental Deterioration Battery	0.71 (-0.34-1.35)	0.67 (-1.04-1.24)	0.76 (-0.55-1.40)	0.365
**Dopaminergic treatment**				
Levodopa treatment, *n (%)*	36 (28.4)	12 (42.9)	24 (24.2)	0.061
Levodopa max daily posology (mg)	300 (300-450)	400 (300-600)	300 (200-450)	0.226
Response to Levodopa, *n (%)*	15 (11.8)	7 (25.0)	8 (8.1)	0.151
**Magnetic resonance imaging:**				
ARWMC score	8 (4-12)	8.5 (4.5-12)	7.5 (4-12)	0.915
Fazekas score	3 (2-4)	2 (1.5-3.5)	3 (2-4)	0.561

Continuous variables are expressed as median (interquartile range).

*Urinary dysfunctions include the following: nycturia, urinary incontinence, pollakiuria, and urinary urgency.

Legend= α-syn-LB: Lewy body-associated α-synuclein seeding activity; ARWMC: Age-Related White Matter Changes; FIM: Functional Independence Measure; iNPH: idiopathic Normal Pressure Hydrocephalus; MDS-UPDRS: Movement Disorder Society-Unified Parkinson's Disease Rating Scale; MMSEc: corrected Mini-Mental State Examination; mRankin: Modified Rankin Scale; MRI: magnetic resonance imaging; TUGT: timed up and go test.

Table S5. Comparisons between Aß+ vs. Aß– subgroups of patients from the Bologna PRO-HYDRO cohort.

	**Aß+**	**Aß-**	***p-value***
**N**	32	94	
**Males**, *n (%)*	22 (68.8)	61 (64.9)	0.830
**Age at disease onset**, *years*	74.0 (70.0-77.0)	72.0 (69.0-76.0)	0.258
**Age at evaluation**, *years*	77.5 (72.5-79.0)	76.0 (72.0-79.0)	0.213
**Disease duration <1 year**, *n (%)*	6 (18.8)	14 (14.9)	0.125
**Symptom of disease onset:**			
Gait disorders, *n (%)*	17 (53.1)	59 (62.8)	
Balance disorders, *n (%)*	3 (9.4)	11 (11.7)	0.107
Urinary dysfunctions, *n (%)*	1 (3.1)	13 (13.8)	
Cognitive impairment, *n (%)*	7 (21.9)	8 (8.5)	
**Symptoms at first evaluation:**			
Gait disorders, *n (%)*	28 (87.5)	90 (95.7)	0.426
Urinary dysfunctions, *n (%)*	21 (65.6)	78 (83.0)	0.560
Cognitive impairment, *n (%)*	23 (71.9)	59 (62.8)	0.108
**Fall in the last year**, n (%)	19 (59.4)	68 (72.3)	0.231
**Number of urinary dysfunctions***	3 (1-4)	3 (2-4)	0.233
**Neurological examination:**			
Partial MDS-UPDRS, motor section score	6.5 (2.0-14.5)	6.0 (4.0-13.0)	0.984
Rest tremor, *n (%)*	6 (19.4)	16 (17.8)	0.196
Gait impairment, *n (%)*	2 (1-2)	2 (1-2)	0.302
Festination, *n (%)*	2 (6.3)	13 (13.8)	0.218
Heel height reduction, *n (%)*	23 (71.9)	66 (70.2)	0.587
Petit-pas gait, *n (%)*	18 (56.3)	43 (45.7)	0.200
Varus, *n (%)*	13 (40.6)	46 (48.9)	0.263
Wide-based gait, *n (%)*	19 (59.4)	58 (61.7)	0.452
Arm swing reduction, *n (%)*	19 (59.4)	51 (54.3)	0.409
Freezing, *n (%)*	2 (6.3)	12 (12.8)	0.253
Start hesitation, *n (%)*	5 (15.6)	7 (7.5)	0.155
**TUGT** *(seconds)*	5.3 (4.3-15.6)	4.7 (3.5-12.9)	0.301
**18-meters walking test:**			
Time 18-m *(seconds)*	28.1 (19.7-38.8)	25.4 (20.4-32.8)	0.818
Gait Speed *(meters for second)*	0.6 (0.5-0.9)	0.7 (0.53-0.9)	0.924
Step length *(meters)*	0.9 (0.5-2.0)	1.4 (0.5-2.2)	0.616
**Number of steps (180° turn)**	4 (3-7)	4 (3-6)	0.663
**Postural Stability**	2 (1-3)	2 (2-3)	0.472
**Scores:**			
iNPH grading scale (max 12)	6 (5-7)	5.5 (4-7)	0.293
Gait status scale (max 16)	5 (3-7)	5 (2-8)	0.692
Tinetti balance	13.5 (10-15)	13 (10-14)	0.257
Tinetti gait	7.5 (5-9)	8 (5-9)	0.813
Tinetti total score (max 28)	21.5 (16-24)	21 (15-24)	0.600
FIM scale (max 126)	105 (96-123)	114.5 (102-121)	0.259
mRankin Scale (max 6)	2 (1-3)	2 (1-3)	0.566
MMSEc (max 30)	24.5(19.3-27.4)	26.4(24.7-27.7)	**0.037**
Brief Mental Deterioration Battery	-0.6 (-2.2-0.8)	0.9 (0.3-1.5)	**0.0003**
**Levodopa treatment**	9 (28.1)	26 (27.7)	0.564
**Magnetic resonance imaging:**			
ARWMC score	6.0 (4.0-11.0)	9.0 (4.0-13.0)	0.161
Fazekas score	2.0 (2.0-3.0)	3.0 (1.0-4.0)	0.326

Data were available for 126 out 127 patients of the Bologna PRO-hydro cohort.

Continuous variables are expressed as median (interquartile range).

*Urinary dysfunctions include the following: nycturia, urinary incontinence, pollakiuria, and urinary urgency.

Legend= ARWMC: Age-Related White Matter Changes; FIM: Functional Independence Measure; iNPH: idiopathic Normal Pressure Hydrocephalus; MDS-UPDRS: Movement Disorder Society-Unified Parkinson's Disease Rating Scale; MMSEc: corrected Mini-Mental State Examination; mRankin: Modified Rankin Scale; TUGT: timed up and go test.

Table S6. Comparisons between responders and non-responders 6 months after surgery.

Bologna iNPH cohort - 6 months follow-up	Non-Responders (n=47)	Responders (n=42)	p-value
**Males**, *n (%)*	37 (78.7)	22 (52.4)	**0.009**
**Age at evaluation**, *years*	76 (72-79)	75 (72-78)	0.599
**Age at disease onset**, *years*	72 (67-75)	73 (69-76)	0.645
**Symptoms at first evaluation**			
Gait disorders, *n (%)*	45 (95.7)	42 (100.0)	0.176
Urinary dysfunctions, *n (%)*	36 (76.6)	35 (30.1)	0.430
Cognitive impairment, *n (%)*	36 (76.6)	25 (59.5)	0.083
**Symptom at disease onset**			0.127
Gait disorders, *n (%)*	29 (61.7)	27 (64.3)	
Balance disorders, *n (%)*	4 (8.5)	7 (16.7)	
Urinary dysfunctions, *n (%)*	3 (6.4)	5 (11.9)	
Cognitive impairment, *n (%)*	11 (23.4)	3 (7.1)	
**Fall in the last year**, *n* (%)	30 (63.8)	35 (83.3)	**0.038**
**Number of urinary dysfunctions**	3 (1-4)	2 (1-4)	0.316
**Neurological examination**			
Rigidity, *n (%)*	30 (63.8)	18 (42.9)	**0.035**
Partial MDS-UPDRS score, motor section	10 (4-15)	5.5 (2-11)	0.071
Gait impairment, *n (%)*	2 (1-2)	2 (1-3)	**0.035**
Festination, *n (%)*	6 (12.8)	5 (11.9)	0.936
Petit-pas gait, *n (%)*	22 (46.8)	23 (54.8)	0.516
Varus, *n (%)*	26 (55.3)	16 (38.1)	0.084
Wide-based gait, *n (%)*	30 (63.8)	24 (57.1)	0.521
Freezing, *n (%)*	7 (14.9)	5 (11.9)	0.651
Start hesitation, *n (%)*	5 (10.6)	6 (14.3)	0.628
**Timed up to go test** *(seconds)*	5.7 (4.0-18.7)	5.0 (4.0-15.0)	0.773
**18-meter walking test**			
Time *(seconds)*	23.3 (18.9-31.0)	28.7 (20.9-34.3)	0.189
Gait Speed *(meters for second)*	0.8 (0.6-0.9)	0.6 (0.5-0.8)	0.123
**Postural Stability**	2 (1-3)	3 (2-3.5)	0.124
**Scores**			
iNPH grading scale (max 12)	6 (4-6)	6 (4-7)	0.724
Gait status scale (max 16)	3.5 (3-6)	6 (4-8)	**0.025**
Tinetti Balance	14 (11-15)	12 (10-14)	0.139
Tinetti Gait	8 (6-9)	7 (4-9)	0.203
Tinetti total score (max 28)	22 (17-24)	20 (14-23)	0.150
mRankin Scale (max 6)	2 (1-3)	3 (2-3)	**0.008**
MMSEc (max 30)	26.3 (24-27.7)	25.8 (23.2-27.3)	0.370
Brief Mental Deterioration Battery	0.53 (-0.59-1.05)	0.88 (0.08-1.47)	0.160
**Levodopa treatment**	19 (40.4)	9 (21.4)	0.069
**ARWMC score - MRI**	8 (6-13)	6.5 (4-10)	0.184
**Fazekas score - MRI**	3 (2-4)	2 (1-3)	0.200
**CSF biomarkers**			
NfL *(pg/ml)*	1063.1 (810.5-1671)	1054.9 (730.5-1274.0)	0.360
total-tau *(pg/ml)*	191.0 (142.0-278.0)	196.0 (135.0-254.0)	0.754
phospho-tau *(pg/ml)*	27.7 (20.1-35.6)	23.0 (19.9-33.0)	0.345
Aβ42/40 ratio	0.9 (0.7-1.0)	0.9 (0.6-1.0)	0.475
Positive α-syn RT-QuIC, *n (%)*	9 (19.2)	9 (21.4)	0.789

Data are expressed as median (interquartile range).

Legend= Aβ: amyloid-beta; α-syn: α-synuclein; ARWMC: Age-Related White Matter Changes; MDS-UPDRS: Movement Disorder Society-sponsored revision of the Unified Parkinson's Disease Rating Scale; MMSEc: corrected Mini-Mental State Examination; mRankin: Modified Rankin Scale; MRI: magnetic resonance imaging; NfL: neurofilament-light chain protein; RT-QuIC: real-time quaking-induced conversion.
